# Dissection of genetic architecture of nine hazardous component traits of mainstream smoke in tobacco (*Nicotiana tabacum* L.)

**DOI:** 10.3389/fpls.2024.1358953

**Published:** 2024-05-08

**Authors:** Manling Xu, Zhijun Tong, Chengting Jin, Qixin Zhang, Feng Lin, Dunhuang Fang, Xuejun Chen, Tianneng Zhu, Xiangyang Lou, Bingguang Xiao, Haiming Xu

**Affiliations:** ^1^ Institute of Bioinformatics and Institute of Crop Science, College of Agriculture and Biotechnology, Zhejiang University, Hangzhou, Zhejiang, China; ^2^ Key Laboratory of Tobacco Biotechnological Breeding, National Tobacco Genetic Engineering Research Center, Yunnan Academy of Tobacco Agricultural Sciences, Kunming, Yunnan, China; ^3^ Department of Biostatistics, University of Florida, Gainesville, FL, United States

**Keywords:** tobacco, mixed linear model, QTL mapping, gene-by-environment interactions, pleiotropy

## Abstract

Tobacco (*Nicotiana tabacum* L.) use is the leading cause of preventable death, due to deleterious chemical components and smoke from tobacco products, and therefore reducing harmful chemical components in tobacco is one of the crucial tobacco breeding targets. However, due to complexity of tobacco smoke and unavailability of high-density genetic maps, the genetic architecture of representative hazardous smoke has not been fully dissected. The present study aimed to explore the genetic architecture of nine hazardous component traits of mainstream smoke through QTL mapping using 271 recombinant inbred lines (RILs) derived from K326 and Y3 in multiple environments. The analysis of genotype and genotype by environment interaction (*GE*) revealed substantially greater heritability over 95% contributed mostly by *GE* interaction effects. We also observed strong genetic correlations among most studied hazardous smoke traits, with the highest correlation coefficient of 0.84 between carbon monoxide and crotonaldehyde. Based on a published high-density genetic map, a total of 19 novel QTLs were detected for eight traits using a full QTL model, of which 17 QTLs showed significant additive effects, six showed significant additive-by-environment interaction effects, and one pair showed significant epistasis-by-environment interaction effect. Bioinformatics analysis of sequence in QTL region predicted six genes as candidates for four traits, of which *Nt21g04598.1*, *Nt21g04600.1*, and *Nt21g04601.1* had pleiotropic effects on PHE and TAR.

## Introduction

Tobacco smoking is the world’s leading cause of avoidable premature mortality (World Health Organization, 2023). Cigarette smoke is a complex, dynamic and reactive mixture that consists of more than 8000 chemical compounds (Rodgman and Green, 2003). When a cigarette is lit, the hot carbonaceous coal within the burning cigarette can reach peak temperatures exceeding 900°C during a puff (McAdam et al., 2016). Adjacent to the hot coal, thermolytic processes (including distillation, pyrolysis and combustion) act on the components of the tobacco to form various smoke constituents, which are released as mainstream smoke (Schwanz et al., 2019).

Due to complexity of tobacco smoke, the mechanism underlying harmfulness of smoke has not been fully understood. Therefore, various lists of toxicants have been proposed in an effort to identify the most relevant constituents responsible for smoking-related diseases (Smith and Hansch, 2000; Liu et al., 2012), including lists of analytes from Hoffman (Hoffmann et al., 1997, 2001), Rodgman and Green (Rodgman and Green, 2003), Talhout (Talhout et al., 2011) and FDA (Food and Drug Administration, 2012). Based on the previous lists and toxicological test methods, a simplified evaluation system was established by Xie et al. (Xie et al., 2009). Xie and his colleagues analyzed 29 hazardous constituents in smoke and four pharmacologic indexes for 163 cigarette samples sold in China. Through statistical analysis, seven smoke constituents, including hydrogen cyanide (HCN), ammonia (NH_3_), phenol (PHE), benzo[α]pyrene (B[a]P), carbon monoxide (CO), crotonaldehyde (CRO), and 4-(methylnitrosamino)-1-(3-pyridyl)-1-butanone (NNK), were selected out to establish a novel hazard index (HI). In addition, given the extreme harm of tar (TAR) in mainstream cigarette smoke to human health, tobacco industry has integrated TAR and HI for comprehensive assessments of hazardous smoke. Efforts are being made to minimize harm, with a focus on tar and the seven representative harmful substances.

There have been reports on the emission of smoke toxicants attributable to several factors such as the variety of chemical compositions in tobacco leaf and the reduction of harmful chemical components within the tobacco leaf is considered as a critical objective in tobacco breeding initiatives (Julio et al., 2006). However, little is currently known about the genetic architecture underlying these smoke-related traits which are considered as quantitative traits. Julio (Julio et al., 2006) was the first to show interest in quantitative trait locus (QTL) mapping and several QTLs of smoke properties (tar, benzo[α]pyrene and CO) were detected in a recombinant inbred line (RIL) population with a partial genetic map. No novel QTL was reported until Tong’s study (Tong et al., 2021) for seven smoke substances, including benzo[a]pyrene, hydrocyanic acid, phenol, carbon monoxide, tar, nicotine and total particle matter, using a high-density genetic map constructed by single nucleotide polymorphisms (SNPs) in RIL population, mapping more QTLs on smoke hazardous components and understanding their interactions with environments is still necessary for efficient molecular genetic improvement of the traits. In this study, based on an integrated high-density linkage map and multi-environment phenotypic data of the RIL population, QTL mapping was conducted for nine hazardous smoke traits; the detected main-effect QTLs, epistastic QTLs and their interactions with environments will provide more insights into the genetic architecture of the traits and greatly facilitate the molecular improvements of breeding low-hazard tobacco varieties.

## Materials and methods

### Plant materials and field trial

The RILs were generated from two elite flue-cured tobacco parents Y3 and K326. Y3 is a backbone cultivated variety that originated from Zimbabwe with elite agronomic traits and complicated parental sources. K326, whose genome has been assembled (Edwards et al., 2017), was introduced from America with high commercial quality and disease resistance but moderate agronomic performance. A total of 274 RILs were employed in this study, consisting of two parents, one F_1_ generation hybrid (YKF_1_; Y3  ×  K326) and 271 F_7_ generation individuals. The materials were planted at Shilin (N: 23.46; E: 103.17) field experiment stations using complete random design with 5 replications, and were cultivated according to local technical measures for quality tobacco production. The eight hazardous substances, including benzo[α]pyrene (B[a]P), carbon monoxide (CO)¸ crotonaldehyde (CRO), hydrogen cyanide (HCN), ammonia (NH_3_), 4-(methylnitrosamino)-1-(3-pyridyl)-1-butanone (NNK), phenol (PHE) and tar (TAR) and were collected in the mainstream smoke of cigarettes produced using tobacco planted at Shilin in 2018, 2019 and 2020; and calculated hazard index (HI). Three combinations of location and year were treated as environments denoted as E1 (2018 Shilin), E2 (2019 Shilin) and E3 (2020 Shilin).

### Measurement and calculation of nine hazard constituents in mainstream smoke

B[a]P was analyzed using the gas chromatography mass spectrometry (GC-MS) method, as described by the Chinese standard method GB/T 21130-2007 (Standardization Administration of China, 2007). CO was determined in the vapor phase using a non-dispersive infrared analyzer, as described by the Chinese standard method GB/T 23356-2009 (Standardization Administration of China, 2009). CRO was analyzed using the high-performance liquid chromatography (HPLC) method, as described by the Chinese tobacco industry standard method YC/T 254–2008 (Standardization Administration of China, 2008a). HCN was quantified using Ion Chromatography, the method as described by the Chinese tobacco industry standard method YC/T 403–2011 (Standardization Administration of China, 2011). NH_3_ was analyzed using Ion Chromatography, the method as described by the Chinese tobacco industry standard method YC/T 377–2017 (Standardization Administration of China, 2017). NNK analysis was carried out using GC-TEA method, as described by the Chinese standard method GB/T 23228-2008 (Standardization Administration of China, 2008b). PHE was analyzed using HPLC, the method as described by the Chinese tobacco industry standard method YC/T 255–2008 (Standardization Administration of China, 2008c). TAR was analyzed using a routine analytical smoking machine, as described by the Chinese standard method GB/T 19609-2004 (Standardization Administration of China, 2004). HI was calculated by the following formula, $HI=\left(\frac{X_{CO}}{14.2}+\frac{X_{HCN}}{146.3}+\frac{X_{NNK}}{5.5}+\frac{X_{NH3}}{8.1}+\frac{X_{B\left[a\right]P}}{10.9}+\frac{X_{PHE}}{17.4}+\frac{X_{CRO}}{18.6}\right)\times 10/7$ (Xie et al., 2009), where $X_{CO}$ represents emission level of CO (mg/cigarette), $X_{HCN}$ for HCN (μg/cigarette), $X_{NNK}$ for NNK (ng/cigarette), $X_{NH3}$ for NH_3_ (μg/cigarette), $X_{B\left[a\right]P}$ for B[a]P (ng/cigarette), $X_{PHE}$ for PHE (μg/cigarette) and $X_{CRO}$ for CRO (μg/cigarette). Three biological replicates were used for each assessment.

### Statistical analysis of phenotypes

Variance components analysis and heritability estimation were performed based on the following linear model,


$$y_{hki}=\mu +{ɡ}_{k}+e_{h}+ɡe_{kh}+\varepsilon_{khi}$$

where $y_{khi}$ is the phenotypic value of the *i*-th replication of the k -th line in the h -th environment; μ is the population mean; $g_{k}$ is the genotypic value of the k -th genotype, random effect, ${ɡ}_{k}∼N\left(0,\sigma_{ɡ}^{2}\right)$ ; $e_{h}$ is the effect of the h -th environment, random, $e_{h}∼N\left(0,\sigma_{e}^{2}\right)$ ; $ɡe_{kh}$ is the interaction effect between the k -th genotype and the h -th environment, random, $ɡe_{kh}∼N\left(0,\sigma_{ɡe}^{2}\right)$ ; $\varepsilon_{khi}$ is the residual effect of the individual, random, $\varepsilon_{khi}∼N\left(0,\sigma_{\varepsilon }^{2}\right)$ .

The *mmer* module of *sommer* R package (Covarrubias-Pazaran, 2016) was applied to estimate the variances of random effects ( ${\hat{\sigma }}_{ɡ}^{2},{\hat{\sigma }}_{e}^{2},\;{\hat{\sigma }}_{ɡe}^{2},{\hat{\sigma }}_{\varepsilon }^{2}$ ) and to predict the random effects by BLUPs (best linear unbiased predictions, ${\hat{g}}_{k},{\hat{e}}_{h},{\hat{ge}}_{kh}$ ) by solving the mixed model equation (MME). Heritability was estimated with the formula $h_{g}^{2}={\hat{\sigma }}_{ɡ}^{2}/\left({\hat{\sigma }}_{ɡ}^{2}+{\hat{\sigma }}_{ɡe}^{2}+{\hat{\sigma }}_{\varepsilon }^{2}\right)$ and $h_{ge}^{2}=\sigma_{ge}^{2}/\left(\sigma_{ɡ}^{2}+\;\sigma_{ɡe}^{2}+\sigma_{\varepsilon }^{2}\right)$ , where ${\hat{\sigma }}_{ɡ}^{2}$ was the estimated genotypic variance, ${\hat{\sigma }}_{ɡe}^{2}$ was the estimated variance due to gene-by-environment interaction, and $\;{\hat{\sigma }}_{\varepsilon }^{2}$ was the estimated residual variance. The *rcorr* module of *Hmisc* R package (https://cran.r-project.org/web/packages/Hmisc/index.html) was employed to calculate Pearson correlation coefficients between six studied traits: (1) phenotypic correlation coefficients with $y_{khi}$ for each environment, respectively; (2) genetic correlation coefficients with ${\hat{y}}_{k}$ . ( ${\hat{y}}_{k}=\hat{\mu }+{\hat{g}}_{k}$ , where ${\hat{y}}_{k}$ is the adjusted genotypic value of the k -th line by the environment effects, $\hat{\mu }$ is the estimated population mean, and ${\hat{g}}_{k}$ is the genotypic value of the k -th line predicted by BLUP).

### Genetic linkage map

The high-resolution linkage map (Tong et al., 2023) that contained 46,324 markers, which were classified into 7,107 bins, a group of markers with least genotype missing rate and same genetic distance, distributed on the 24 linkage groups (LGs) and covered 3334.88 cM with an average genetic distance of 0.469 cM, was employed in this study.

### Genetic model and statistical methods for QTL mapping

A QTL full model was adopted for modeling the genetic architecture of complex traits from multi-environment trials, which includes additive effect (*a*) of each QTL, additive-by-additive epistatic effect (*aa*) of each pair of epistatic QTL, treated as fixed effects, and their corresponding environment interaction effects (*ae* and *aae*) as random effects. Suppose a trait is controlled by s segregating QTLs, of which t pairs of QTLs are involved in epistasis. Then, the phenotypic value of the *m*-th replication of the k -th genotype in the h -th environment ( $y_{hkm}$ ) can be expressed by the following mixed linear model (Yang et al., 2007),


$$\begin{array}{c} y_{hkm}=\mu +\sum_{i=1}^{s}a_{i}x_{ik}+\sum_{\begin{array}{c} i,j\in \left\{1,2\ldots ,s\right\}\\ i\neq j \end{array}}^{t}aa_{ij}x_{ik}x_{jk}+e_{h}+\sum_{i=1}^{s}ae_{hi}u_{hik}+\sum_{\begin{array}{c} i,j\in \left\{1,2\ldots ,s\right\}\\ i\neq j \end{array}}^{t}aae_{hij}u_{hijk}+\epsilon_{hkm} \end{array}$$

where, μ is the population mean; $a_{i}$ is the additive effect of the i -th QTL with indicator variable $x_{ik}$ , fixed effect; $aa_{ij}$ is the additive-by-additive epistatic effect of the i -th QTL and the j -th QTL with indicator variable $x_{ik}x_{jk}$ , fixed effect; $e_{h}$ is the effect of the h -th environment, random effect, $e_{h}∼\left(0,\sigma_{E}^{2}\right)$ ; $ae_{hi}$ is the additive-by-environment interaction effect of the i -th QTL and the h -th environment with observation $u_{hik}$ ( $=x_{ik}$ ), random effect, $ae_{hi}∼\left(0,\;\sigma_{A_{i}E}^{2}\right)$ ; $aae_{hij}$ is the interaction effect of the $aa_{ij}$ and the h -th environment with observation $u_{hijk}$ ( $=x_{ik}x_{jk}$ ), random effect, $aae_{hij}∼\left(0,\sigma_{AA_{ij}E}^{2}\right)$ ; and $\epsilon_{hkm}$ is the residual effect of the individual, random, $\epsilon_{hkm}∼\left(0,\;\sigma_{\epsilon }^{2}\right)$ .

*QTLNetwork* 2.0 software were employed to detect QTLs by the mixed-linear-model-based composite interval mapping (MCIM) method (Yang et al., 2008). One- and two-dimensional genome scans for QTLs were performed with configurations of 10 cM testing window, 1 cM walking step and 10 cM filtration window size. To control the experiment-wise type I error rate, a critical *F*-value based on the Henderson III method was determined by the permutation test with 1,000 times for each tested locus at the significance level of 0.05. Based on the significant QTL, a QTL full model was established and used to estimate each parameter based on the samples generated by Markov chain Monte Carlo (MCMC) with 20,000 Gibbs sampler iterations.

### Candidate genes prediction

The physical positions of the marker interval with QTL were determined using Nucleotide BLAST module of NCBI (https://blast.ncbi.nlm.nih.gov/Blast.cgi), which utilized sequence information of two adjacent bin markers in the linkage map. Variants including SNPs and Indels located within the QTL regions were selected for subsequent filtration. These variants were annotated by software SnpEff (http://pcingola.github.io/SnpEff/) based on K326 reference genome (https://solgenomics.net/ftp/genomes/Nicotiana_tabacum/edwards_et_al_2017/assembly/Nitab-v4.5_genome_Chr_Edwards2017.fasta ), and those showing HIGH or MODERATE impact on related protein effectiveness were retained, according to annotation results. Then, the eligible variants with *P* value less than 0.05 were identified by performing single-marker regressions with ${\hat{y}}_{k}$ as response variable (as mentioned in the **Statistical analysis of phenotypes**). Before performing the enrichment analysis of genes with variants above, the protein sequences of the K326 reference genome (https://solgenomics.net/ftp/genomes/Nicotiana_tabacum/edwards_et_al_2017/annotation/Nitab-v4.5_proteins_Edwards2017.fasta) should be uploaded to the eggNOG-mapper website (http://eggnog-mapper.embl.de/) for functional annotation. Gene Ontology (GO) and KEGG pathway enrichment analyses were carried out using the *clusterProfiler* R package (https://github.com/YuLab-SMU/clusterProfiler) as criteria for predicting candidate genes.

## Results

### Phenotypic performance of nine smoke-related traits

**Figure 1:**
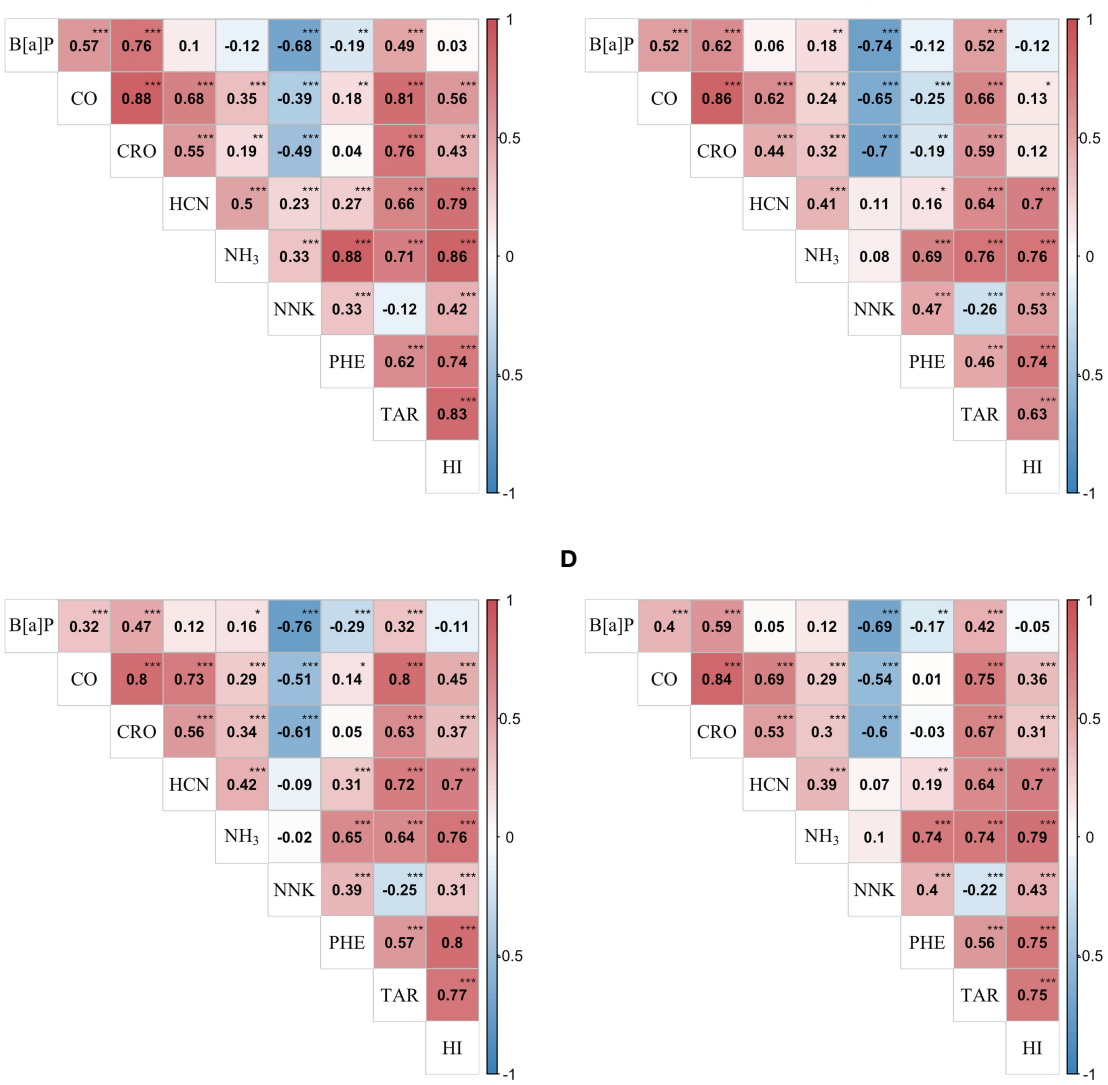
Phenotypic and genetic correlations between studied traits in the RIL population. Heat map **(A–C)** showed phenotypic correlation coefficients between nine traits in E1 (2018 Shilin), E2 (2019 Shilin), E3 (2020 Shilin) in turn, the heat map **(D)** showed genetic correlation coefficients between nine traits. *, **, *** denote significance level at 0.05, 0.01 and 0.001, respectively. Traits abbreviations are same as those in **Table 1**.

For the nine smoke-related traits, the estimated heritability of genetic effects $h_{ɡ}^{2}$ ranged from 11.03% for CRO to 20.00% for CO, displaying a limited stability across environments for these traits (**Table 1**). Furthermore, gene-by-environment effects were found to contribute significantly more to phenotypic variation with most estimated interaction heritability ( ${\hat{h}}_{ɡe}^{2}$ ) nearly over 80%, indicating selection of these traits should design specific strategy for different environment conditions. Most phenotypic correlations of the traits were positive and reached statistical significance ( α=0.05 ), for example, with coefficients over 0.8 between CO and CRO in three environments (**Figures 1A-C**). Nevertheless, strong negative correlations were observed between NNK and other three constituents: B[a]P, CO, and CRO, while, weak negative correlations between B[a]P and PHE, and between NNK and TAR. In general, the phenotypic correlations in three environments showed similar pattern as well as the genetic correlations (**Figure 1D**), indicating a strong and reliable underlying genetic relationship among these traits that make further exploration necessary. However, inconsistencies in the performance of the traits across three environments could be observed, with E2 exhibiting a higher frequency of exceptions. For instance, the phenotypic correlations between CO and PHE were significantly positive in E1 (**Figure 1A**) and E3 (**Figure 1C**), but significantly negative in E2 (**Figure 1B**). The genetic correlation coefficients, calculated using the estimated genotypic values, exhibited the highest correlation of 0.84 between CO and CRO, followed by 0.79 between NH_3_ and HI, and several other strong correlations above 0.7, such as NH_3_ and PHE (**Figure 1D**). The existence of high underlying genetic correlations between these traits might help to explain their strong phenotypic correlations. Further, correlations between HI and the other traits were all displayed significantly positive except B[a]P, which is consistent with the fact that HI was a composite index comprising seven representative smoke traits.

**Table 1:** Variance components analysis and estimated heritability (%) of genetic effects for smoke-related traits.

Trait ^a^	Variance Components ( $\sigma^{2})$ ^b^				$h_{g}^{2}$ ^c^ (%)	$h_{ge}^{2}$ ^d^ (%)
	$\sigma_{g}^{2}$	$\sigma_{e}^{2}$	$\sigma_{ge}^{2}$	$\sigma_{\varepsilon }^{2}$		
B[a]P	0.22	0.76	1.36	0.05	13.50	83.44
CO	0.64	5.74	2.46	0.10	20.00	76.88
CRO	1.01	15.03	7.78	0.37	11.03	84.93
HCN	49.69	343.76	347.57	10.61	12.18	85.22
NH_3_	0.23	1.03	1.78	0.05	11.17	86.41
NNK	0.79	6.79	4.36	0.12	14.99	82.73
PHE	1.38	15.09	8.58	0.27	13.49	83.87
TAR	0.31	1.28	1.51	0.04	16.67	81.18
HI	0.11	0.78	0.75	0.02	12.50	85.23

a Trait abbreviation: B[a]P for benzo[α]pyrene; CO for carbon monoxide; CRO for crotonaldehyde; HCN for hydrogen cyanide; NH_3_ for ammonia; NNK for 4-(methylnitrosamino)-1-(3-pyridyl)-1-butanone; PHE for phenol; TAR for tar; HI for hazard index of mainstream smoke.

b Variance components ( $\sigma^{2})$ : $\sigma_{ɡ}^{2}$ is for genotypic variance, $\;\sigma_{e}^{2}$ for environmental variance, $\sigma_{ɡe}^{2}$ for gene-by-environment interaction variance, $\;\sigma_{\varepsilon }^{2}$ for error variance.

c $h_{g}^{2}=\sigma_{ɡ}^{2}/\left(\sigma_{ɡ}^{2}+\;\sigma_{ɡe}^{2}+\sigma_{\varepsilon }^{2}\right)$ .

d $h_{ge}^{2}=\sigma_{ɡe}^{2}/\left(\sigma_{ɡ}^{2}+\;\sigma_{ɡe}^{2}+\sigma_{\varepsilon }^{2}\right)$ .

### Additive and additive-by-environment interaction effects

A total of 19 QTLs were identified responsible for the nine smoke-related traits. Among them, NH_3_ had four QTLs; CO, NNK, PHE and TAR each had three QTLs; CRO, HCN and HI each only detected one QTL (**Table 2**). These QTLs distributed on seven linkage groups (LGs), with LG15 containing the highest number of QTLs (7 QTLs), followed by LG6 (6 QTLs) and LG1 (2 QTLs), while each of LG4, LG5, LG10 and LG19 only harbored one QTL.

**Table 2:** QTLs detected for smoke-related traits in RIL population.

Trait	QTL ^a^	M- ^b^	M+ ^b^	Position	Support interval ^c^	Type ^d^
				(cM)	(cM)	
CO	*qCO6*	SNP_0000296_289859	SNP_0062500_2607	168.2	166.6-169.1	A
	*qCO15*	SNP_0003624_262762	SNP_0012350_25875	71.3	70.0-72.2	A, AE
	*qCO19*	SNP_0447938_215	SNP_0003870_3280	91.7	90.0-92.6	A
CRO	*qCRO4*	SNP_0001337_231331	SNP_0001310_519614	95.4	93.4-98.4	A, AE
HCN	*qHCN6*	SNP_0000117_506521	SNP_0012136_2575	157	156.6-157.3	A
NH_3_	*qNH_3_5*	InDel_0007135_115136	SNP_0000422_1021836	86.9	85.1-87.7	AAE
	*qNH_3_10*	SNP_0000443_335917	InDel_0026440_4162	16.1	13.5-17.8	AAE
	*qNH_3_15-1*	SNP_0541841_487	SNP_0004003_99097	60.6	59.0-60.8	A
	*qNH_3_15-2*	SNP_0002539_133	SNP_0000535_1549	118.5	116.9-120.2	A
NNK	*qNNK6*	PT61401	SNP_0243711_753	14	13.3-15.5	A, AE
	*qNNK15-1*	SNP_0005871_32659	SNP_0009221_79474	70.7	70.3-72.2	A, AE
	*qNNK15-2*	SNP_0072565_795	InDel_0109986_220	123.1	121.3-124.2	A
PHE	*qPHE1*	PT61201	SNP_0912209_288	117.7	117.0-118.1	A, AE
	*qPHE6*	SNP_0002499_215080	SNP_0011326_16206	0	0.0-2.2	A
	*qPHE15*	SNP_0003210_25891	SNP_0710272_305	65.2	64.6-65.7	A, AE
TAR	*qTAR1*	PT61201	SNP_0912209_288	117.7	117.6-117.9	A
	*qTAR6*	SNP_0002499_215080	SNP_0011326_16206	0	0.0-1.4	A
	*qTAR15*	SNP_0002539_133	SNP_0000535_1549	118.5	117.8-121.3	A
HI	*qHI6*	SNP_0065615_277	SNP_0241015_536	11.8	11.6-12.2	A

a QTL: named in the form of “q”,”Trait”,”LG”,”-Rank”, for example, qCO6 denotes a QTL of CO which is the first QTL on the LG6;

b M-, M+: flanking markers, of which markers whose name begin with TM and PT denote SSR.

c Support interval of a QTL: determined by following strategy: firstly, search the first left and right tested positions whose P-values increase to ten times of that of the QTL, then select their nearest markers for the support interval of the QTL.

d Type: A, AE and AAE denote QTL with additive effects, additive-by-environment interaction effects and epistasis-by-environment interaction effects, respectively.

^*^, ^**^, ^***^ denote significance level at 0.05, 0.01 and 0.001, respectively. Abbreviations of traits are same as those in **Table 1**.

It is noteworthy that *qPHE1* and *qTAR1* located in the same marker interval with flanking marker PT61201 and SNP_0912209_288, similarly, *qPHE6* and *qTAR6* in the same marker interval ranged by SNP_0002499_215080 and SNP_0011326_16206, *qNH_3_15-2* and *qTAR15* in the same interval ranged by SNP_0002539_133 and SNP_0000535_1549 (**Table 2**). These co-location indicated the potential pleiotropic effect of QTL on PHE and TAR or on NH_3_ and TAR, which requires further investigation for revealing the molecular mechanism of genetic correlation between traits. Our inference on existence of pleiotropic QTL was enhanced by the significant and relatively high genetic correlations estimated between the studied traits (**Figure 1**), which were 0.74 between NH_3_ and TAR, and 0.56 between PHE and TAR as we mentioned above.

Totally, 17 QTLs with additive (*a*) main effects were detected for nine traits, of which six QTLs also showed additive-by-environment interaction (*ae*) effects (**Table 3**). Most of the QTLs exhibited small additive effects, which were regarded as minor-effect QTLs and accounted for approximately 2% phenotypic variance. The average proportion of phenotypic variance explained by individual QTL ( $h_{a}^{2}$ ) was 2.28%. Moreover, around 70% of all QTLs each explained less than 3% of the phenotypic variance, the rest QTLs taking relatively larger effects on five traits, *qHCN6* ( $h_{a}^{2}$ =4.99%), *qHI6* ( $h_{a}^{2}$ =3.39%), *qTAR6* ( $h_{a}^{2}$ =3.14%), *qCO15* ( $h_{a}^{2}$ =3.22%), *qNH_3_15-2* ( $h_{a}^{2}$ =3.21%), located mainly on LG6 and LG15. Most QTLs, whose homozygous genotypes of the alleles from the parent K326 (*QQ*), contributed positive additive effects (i.e., increasing the trait value) (**Table 4**); in contrast, the other QTL genotype with alleles from the parent Y3 (*qq*) contributed negative additive effects.

**Table 3:** Effects and the proportion of phenotypic variance explained by QTL.

Trait	QTL	*a* ^a^		*ae_1_*	*ae* ^b^	*ae_3_*	PVE(%) ^e^		
					*ae_2_*		A	AE	
CO	*qCO6*	0.4814^***^					1.55	0	
	*qCO15*	-0.4127^***^		0.3316^**^		-0.3225^**^	3.22	1.13	
	*qCO19*	0.3048^***^					1.06	0	
CRO	*qCRO4*	-0.4698^***^		0.4392^*^		-0.3724^*^	1.04	0.80	
HCN	*qHCN6*	7.1922^***^					4.99	0	
NH_3_	*qNH_3_15-1*	0.3234^***^					2.84	0	
	*qNH_3_15-2*	-0.3186^***^					3.21	0	
NNK	*qNNK6*	0.3407^***^		-0.3513^**^	0.4153^**^		0.80	1.17	
	*qNNK15-1*	0.3838^***^		-0.3424^*^		0.4939^***^	2.05	1.38	
	*qNNK15-2*	0.5128^***^					2.51	0	
PHE	*qPHE1*	0.4886^***^				0.4876^*^	1.03	0.70	
	*qPHE6*	0.7360^***^					1.65	0	
	*qPHE15*	0.5749^***^		-0.5163^**^			1.49	0.89	
TAR	*qTAR1*	0.2458^***^					2.01	0	
	*qTAR6*	0.3018^***^					3.14	0	
	*qTAR15*	-0.2386^***^					2.85	0	
HI	*qHI6*	0.2215^***^					3.39	0	

Trait	QTL*_i_*	QTL*_j_*	*aa* ^c^	*aae* ^d^			PVE(%) ^e^		
				*aae*_1_	*aae*_2_	*aae*_3_	AA		AAE
NH_3_	*qNH_3_5*	*qNH_3_10*		0.2417^**^	-0.1732^*^		0		1.51

a a: additive effect.

b ae: additive-by-environment interaction effects, of which ae_1_ denotes the interactions between a and environment E1.

c aa: additive-by-additive epistatic effect.

d aae: epistasis-by-environment interaction effects, of which aae_1_ denotes the interaction between aa and environment E1.

e PVE(%), the proportion of phenotypic variance explained; PVE(A), the proportion of phenotypic variance explained by the additive QTL; PVE(AE), the proportion of phenotypic variance explained by the additive-by-environment interaction; PVE(AA), the proportion of phenotypic variance explained by the additive-additive epistatic effects; PVE(AAE), the proportion of phenotypic variance explained by the epistasis-by-environment interaction.

^*^, ^**^, ^***^ denote significance level at 0.05, 0.01 and 0.001, respectively. Abbreviations of traits are same as those in **Table 1**.

**Table 4:** Superior lines predicted by full genetic model for smoke-related traits.

Trait	QTL	GSL(-) ^a^	SL(-)1 ^b^	SL(-)2	SL(-)3
CO	*qCO6*	*qq*	*qq*	*qq*	*qq*
	*qCO15*	*QQ*	*QQ*	*QQ*	*QQ*
	*qCO19*	*qq*	*qq*	*qq*	*qq*
CRO	*qCRO4*	*QQ*	*QQ*	*QQ*	*QQ*
HCN	*qHCN6*	*qq*	*qq*	*qq*	*qq*
NH_3_	*qNH_3_5*	*QQ/qq*	*qq*	*QQ*	*QQ/qq*
	*qNH_3_10*	*QQ/qq*	*QQ*	*QQ*	*QQ/qq*
	*qNH_3_15-1*	*qq*	*qq*	*qq*	*qq*
	*qNH_3_15-2*	*QQ*	*QQ*	*QQ*	*QQ*
NNK	*qNNK6*	*qq*	*QQ*	*qq*	*qq*
	*qNNK15-1*	*qq*	*qq*	*qq*	*qq*
	*qNNK15-2*	*qq*	*qq*	*qq*	*qq*
PHE	*qPHE1*	*qq*	*qq*	*qq*	*qq*
	*qPHE6*	*qq*	*qq*	*qq*	*qq*
	*qPHE15*	*qq*	*qq*	*qq*	*qq*
TAR	*qTAR1*	*qq*	*qq*	*qq*	*qq*
	*qTAR6*	*qq*	*qq*	*qq*	*qq*
	*qTAR15*	*QQ*	*QQ*	*QQ*	*QQ*
HI	*qHI6*	*qq*	*qq*	*qq*	*qq*

a GSL(-): general superior line with minimized trait value for three environments;

b SL(-)1, SL (-)2, SL (-)3: superior line with minimized trait value for E1 (2018 Shilin), E2 (2019 Shilin), E3 (2020 Shilin), respectively; QQ: homozygous genotype of the allele from the parent K326 (P1), qq: homozygous genotype of the allele from the parent Y3 (P2), QQ/qq: indicate the genotype could be QQ or qq.

Abbreviations of traits are the same as **Table 1**.

Significant additive by environment interaction effects (*ae*) were found for less than half of QTLs, and their contributions to phenotypic variation ( $h_{ae}^{2}$ ) were around 1%, with most being lower than their corresponding additive effects. However, there was an exception that the interaction between *qNNK6* and two environments (E1, E2) accounted for 1.17% of the phenotypic variance, which was higher than 0.80% explained by corresponding additive effects. Moreover, the *ae* effects of QTL could take same or opposite effect direction as their main effects (*a*), and also showed different effect direction across environments. However, the *ae* effects of different QTLs for same trait may show consistent effect direction in same environment. For example, the *ae* effects of *qNNK6* and *qNNK15-1* both contributed negative effects in E1. It is widely recognized that QTLs with no significant *ae* effects have crucial application potential in breeding new varieties with strong environment adaptability. Of significance, the absence of *ae* effects of QTLs for HCN, NH_3_, TAR, and HI suggested that these four traits may exhibit stable performance across various environments.

### Additive-by-additive epistasis and epistasis-by-environment interaction effects

Two-dimensional genome scan found a digenic epistatic QTL pair (*qNH_3_5- qNH_3_10*) with epistasis-by-environment interaction effects (*aae*) for NH_3_, but no paired epistatic QTLs for other traits (**Table 3**). This epistatic QTLs didn’t contribute additive-additive epistatic effects (*aa*), only positive *aae* effects in E1 and negative in E2, accounting for 1.51% of total phenotypic variation. Additionally, the epistatic QTL pair *qNH_3_5* on LG5 and *qNH_3_10* on LG10, each had no individual additive effect.

Overall, environmental effect (*E*) explained the largest part of the phenotypic variance which ranged from 26.23% to 53.83%, followed by genetic main effect (*G*) and gene-by-environment interaction effect (*GE*) (**Supplementary Table S1**). For all traits, the *G* effects, which were entirely composed of additive effects of one to three QTLs, explained 1.04% to 8.00% of the phenotypic variance. Besides, for CO, CRO, NH_3_, NNK, PHE, the contribution of *GE* to the total variation was small, with some in the form of *ae* effects and others in the form of *aae* effects. In summary, our analysis showed that these traits were primarily controlled by a single major gene or polygenes.

### Prediction of superior genotype for smoke-related traits

To explore the potential of the identified QTLs in improving smoke-related traits through genetic and molecular manipulation, we undertook QTL genotype design and assessed the trait values potentially achieved by the general superior homozygous line (GSL(-)) and the superior homozygous line for each year (SL(-)) based on the genetic effects of these QTLs. Notably, for the HCN, TAR, and HI, there are no QTL involved in interaction with environment, the designed superior genotypes GSL(-) and SL(-) of each trait remained consistent across all environments (**Table 4**). On the CO, CRO, NH_3_, NNK, and PHE, although they were influenced by gene-environment effects, their superior lines (GSL, SL) have identical homozygous genotypes for each trait across three environments, respectively, except the SL(-)1 with *QQ* at *qNNK6* for NNK. In particular, the superior lines of HCN, NNK and PHE were constructed by all homozygous genotypes with alleles from the parent Y3 (*qq*) at QTLs except *qNNK6* for NNK, which indicated that the pyramid of genes from Y3 at these QTLs can effectively decrease the levels of hazardous smoke constituents, HCN, NNK, and PHE. Meanwhile, this advantageous genotype remained valid under all environments. This discovery provided valuable indication for efficient simultaneous improvements of HCN, NNK, and PHE by utilization of elite genes in Y3.

### Enrichment analysis and prediction of candidate gene for smoke-related traits

Annotation of variants (SNP/Indel) was conducted using *SnpEff* based on the K326 reference genome, and 559,513 variants were identified within the putative physical positions of the 17 additive-QTL regions. Among these, only variants with a HIGH or MODERATE impact on protein function were retained, as they were considered potentially functional in candidate genes, as a result, 5,268 variants were selected in total. Then simple regression analysis of the predicted genetic values on these variants was performed, and 600 significant variants in 76 genes were selected ( P<0.05 ) (**Supplementary Table S2**). These prioritized genes demonstrated enrichment in five GO biological processes, four GO molecular functions, and two KEGG pathways. Specifically, the enrichment was observed in cellular processes (biological process), catalytic activity (molecular function), and signaling molecules metabolic pathways (**Table 5**). For instance, “callose deposition in cell wall”, “polysaccharide localization” and “callose localization” were associated with carbohydrate biosynthesis.

**Table 5:** Significantly enriched Gene Ontology (GO) terms and enriched KEGG pathway terms of potential genes.

ID	Enrichment analysis ^a^	Description	Trait	geneID
GO:0052386	GO_BP	Cell wall thickening	PHE, TAR	*Nt21g04600.1/Nt21g04601.1*
GO:0052543	GO_BP	Callose deposition in cell wall	PHE, TAR	*Nt21g04600.1/Nt21g04601.1*
GO:0033037	GO_BP	Polysaccharide localization	PHE, TAR	*Nt21g04600.1/Nt21g04601.1*
GO:0052545	GO_BP	Callose localization	PHE, TAR	*Nt21g04600.1/Nt21g04601.1*
GO:0048658	GO_BP	Anther wall tapetum development	PHE, TAR	*Nt21g04600.1/Nt21g04601.1*
GO:0008081	GO_MF	Phosphoric diester hydrolase activity	CO, PHE, TAR	*Nt20g03473.1/Nt21g04598.1*
GO:0004620	GO_MF	Phospholipase activity	CO, PHE, TAR	*Nt20g03473.1/Nt21g04598.1*
GO:0016298	GO_MF	Lipase activity	CO, PHE, TAR	*Nt20g03473.1/Nt21g04598.1*
GO:0034480	GO_MF	Phosphatidylcholine phospholipase C activity	PHE, TAR	*Nt21g04598.1*
map00562	KEGG pathway	Inositol phosphate metabolism	NNK, PHE, TAR	*Nt16g00273.1/Nt16g00284.1/Nt21g04598.1*
map00565	KEGG pathway	Ether lipid metabolism	NNK, PHE, TAR	*Nt20g03473.1/Nt21g04598.1*

a Enrichment analysis: Gene Ontology (GO) enrichment analysis, including biological process (BP), molecular function (MF) and cellular component (CC);

Abbreviations of traits are the same as **Table 1**.

The genes *Nt21g04598.1*, *Nt21g04600.1* and *Nt21g04601.1* were predicted as potential pleiotropic candidate genes for *qPHE1* (PHE) and *qTAR1* (TAR), BLASTP results indicated their putative functions. *Nt21g04598.1* was shown to encode a protein highly homologous to non-specific phospholipase C4 in *Arabidopsis thaliana*, which functioned as plasma membrane bound and promoted tolerance to phosphate deficiency and hyperosmotic stress (Nakamura et al., 2005; Peters et al., 2010; Wimalasekera et al., 2010; Kocourková et al., 2011; Pejchar et al., 2015; Yang et al., 2021). *Nt21g04600.1* and *Nt21g04601.1* were predicted to code a protein with high homology to transcription factor bHLH91 in *Arabidopsis thaliana*, which regulated the transcriptional expression, thereby regulating the plant’s adaptive responses (Qian et al., 2021). The gene *Nt20g03473.1* was pinpointed as a candidate gene for *qCO6* (CO), which encoded a protein highly homologous to phospholipase D (PLD) delta in *Arabidopsis thaliana*. PLD delta has been proposed to play a role in many cellular processes such as signal transduction, membrane trafficking, cytoskeletal rearrangements, and membrane degradation (Distéfano et al., 2012; Guo et al., 2012; Uraji et al., 2012; Jia et al., 2013); for example, it was involved in H_2_O_2_ and abscisic acid (ABA)-induced stomatal closure, nitric oxide (NO) signaling and ABA-promoted senescence. Moreover, gene *Nt16g00273.1* and *Nt16g00284.1* have been identified as candidate genes for *qNNK6* (NNK), which have been predicted to encode uncharacterized serine-rich protein C215.13 and type II inositol polyphosphate 5-phosphatase 15 isoform X2 in *Nicotiana tabacum*, respectively, awaiting further annotation in the future.

## Discussion

In comparison to traditional technologies used to reduce harmful ingredients in cigarette smoke, targeting the molecular mechanisms underlying the production of hazardous substances would be an innovative approach to improve safety and quality. Julio et al. (Julio et al., 2006) was the first to apply QTL analysis to explore smoke toxicants. They constructed a partial genetic map using 138 low-throughput markers, including amplified fragment length polymorphism (AFLP), inter simple sequence repeat (ISSR), sequence specific amplified polymorphism (SSAP) and sequence characterized amplified region (SCAR), which were assigned to 18 linkage groups. Then, a total of five QTLs were identified for TAR, CO, and B[a]P in a RIL population. Furthermore, Tong (Tong et al., 2021) conducted QTL studies using a high-density genetic map with 45,081 SNPs, which was constructed by whole-genome sequencing data of a tobacco population of 274 individuals. They detected several major QTLs of PHE, CO and TAR distributed in LG6 from 123.28 to 158.72 cM, and the close linkage of these QTLs were in accord with the strong positive correlations among these traits. Similarly, our study also detected some QTLs of PHE and TAR in the same region, pleiotropic effects or linkage of which may lead to high genetic correlation between the traits.

Compared with previous QTL studies on hazardous smoke-related traits, our study possesses following advantages. First, we utilized a up-to-date published integrated linkage map with 46,324 polymorphic markers, including SNPs, Indels and SSRs, distributed on 24 linkage groups and covered 3334.88 cM with an average genetic distance of 0.469 cM (Tong et al., 2023). This high-resolution linkage map represents the most comprehensive map of tobacco to date. Second, we employed a QTL full model with effects of additive, additive-additive epistasis, and their interaction with environments, which is more rational to depict the genetic properties of quantitative traits where gene-gene and gene-environment interaction are mostly involved. As a result, we identified a total of 19 QTLs for the studied hazardous smoke-related traits, of which 17 QTLs showed significant additive effects, six showed significant additive-by-environment interaction effects, one pair showed significant epistasis-by-environment interaction effects for NH_3_. Notably, only one QTL was detected for CRO, HCN and HI, but more QTLs for CO, NH_3_, PHE and TAR, respectively, probably due to relatively lower general heritability ( $h_{g}^{2}$ ) and interaction heritability ( $h_{ge}^{2}$ ) of CRO, HCN and HI.

Besides, we included the comprehensive index HI in QTL analysis to explore whether any pleiotropic QTLs could be detected with the eight direct smoke-related traits. It turned out that *qHI6* was found to be located on the same linkage group as *qNNK6*, *qCO6*, *qHCN6* and *qPHE6*. This is in concert with the high genetic correlations over 0.7 between HI and HCN, NH_3_ and PHE. In addition, considering of no QTL detected for B[a]P and its relatively small genotypic variance and genotype by environment interaction variance, we inferred that the B[a]P may be controlled by minor-effect QTLs which couldn’t be detected by the program because of too small effect magnitude, unlike major QTLs with relatively large effects that are more likely to be detected (Heffner et al., 2009; Beavis, 2019).

Our investigation pinpointed the *qPHE1* and the *qTAR1* were co-localized in the same chromosome, demarcated by the genetic markers PT61201 and SNP_0912209_288. Similarly, *qPHE6* and *qTAR6* were found to co-localize in another region, flanked by SNP_0002499_215080 and SNP_0011326_16206. These findings suggested the possibility that pleiotropic genes, may be nested within these genomic segments. Furthermore, our hypothesis on existence of pleiotropic gene was reinforced by the substantial genetic correlation with coefficient of 0.56 between the corresponding traits, PHE and TAR. The strong and stable trait correlation across environments further indicated the existence of shared pleiotropic candidate genes in these identified genomic regions. With subsequent comprehensive bioinformatics analyses, *Nt21g04598.1*, *Nt21g04600.1* and *Nt21g04601.1* were anchored at and predicted as pleiotropic candidate genes for *qPHE1* and *qTAR1*. According to the results of NCBI-BLASTP, *Nt21g04598.1* was predicted to encode a protein highly homologous to non-specific phospholipase C4 in *Arabidopsis thaliana*, *Nt21g04600.1* and *Nt21g04601.1* were predicted to code a protein with high homology to transcription factor bHLH91 in *Arabidopsis thaliana*. Coupled with results of GO and KEGG enrichment analyses, we speculated that these candidate genes might be involved in the signaling or regulatory processes of carbohydrate biosynthesis and transformation, leading to different performances of PHE and TAR.

In our study, identifying candidate genes relied on the rough physical positions of QTL intervals, which might encompass hundreds or thousands of genes. In other words, distinguishing target trait genes from annotated genes within a QTL remains challenging for primary mapping populations such as RILs used in our study. Therefore, fine mapping using secondary mapping populations (e.g., SSSLs, NILs, CSSLs), are needed to narrow down target QTLs, eliminating genetic background interference for accurate candidate gene prediction. Additionally, further studies are needed for functional validation of these candidate genes with cutting-edge molecular biology techniques on the levels of gene expressions, proteins and metabolites, so that, the pleiotropic genes could be effectively used in synchronous improvement of PHE and TAR.

## Data availability statement

The original contributions presented in the study are included in the article/**Supplementary Material**. Further inquiries can be directed to the corresponding authors.

## Author contributions

MX: Data curation, Writing – original draft. ZT: Conceptualization, Funding acquisition, Investigation, Project administration, Supervision, Writing – original draft. CJ: Data curation, Validation, Writing – original draft. QZ: Data curation, Methodology, Software, Writing – original draft. FL: Methodology, Software, Writing – original draft. DF: Data curation, Investigation, Writing – original draft. XC: Data curation, Investigation, Writing – original draft. TZ: Methodology, Software, Validation, Writing – original draft. XL: Methodology, Writing – review & editing. BX: Conceptualization, Funding acquisition, Project administration, Resources, Writing – review & editing. HX: Conceptualization, Methodology, Project administration, Supervision, Validation, Writing – review & editing.
